# The electronic Rothamsted Archive (e-RA), an online resource for data from the Rothamsted long-term experiments

**DOI:** 10.1038/sdata.2018.72

**Published:** 2018-05-15

**Authors:** Sarah A. M. Perryman, Nathalie I. D. Castells-Brooke, Margaret J. Glendining, Keith W. T. Goulding, Malcolm J. Hawkesford, Andy J. Macdonald, Richard J. Ostler, Paul R. Poulton, Christopher J. Rawlings, Tony Scott, Paul J. Verrier

**Affiliations:** 1 Computational and Analytical Sciences, Rothamsted Research, Harpenden, Hertfordshire AL5 2JQ, UK; 2 Sustainable Agriculture Sciences, Rothamsted Research, Harpenden, Hertfordshire AL5 2JQ, UK; 3 Plant Sciences, Rothamsted Research, Harpenden, Hertfordshire, Hertfordshire AL5 2JQ, UK; 4 Biointeractions and Crop Protection, Rothamsted Research, Harpenden, Hertfordshire AL5 2JQ, UK

**Keywords:** Agroecology, Databases

## Abstract

The electronic Rothamsted Archive, e-RA (www.era.rothamsted.ac.uk) provides a permanent managed database to both securely store and disseminate data from Rothamsted Research’s long-term field experiments (since 1843) and meteorological stations (since 1853). Both historical and contemporary data are made available via this online database which provides the scientific community with access to a unique continuous record of agricultural experiments and weather measured since the mid-19^th^ century. Qualitative information, such as treatment and management practices, plans and soil information, accompanies the data and are made available on the e-RA website. e-RA was released externally to the wider scientific community in 2013 and this paper describes its development, content, curation and the access process for data users. Case studies illustrate the diverse applications of the data, including its original intended purposes and recent unforeseen applications. Usage monitoring demonstrates the data are of increasing interest. Future developments, including adopting FAIR data principles, are proposed as the resource is increasingly recognised as a unique archive of data relevant to sustainable agriculture, agroecology and the environment.

## Introduction

Sir John Bennet Lawes (1814–1900) was a Victorian entrepreneur; a businessman, farmer and scientist who owned the estate of Rothamsted Manor in Harpenden, Hertfordshire, UK. Joseph Henry Gilbert (1817–1901) was a chemist appointed in 1843 to manage the field experiments Lawes had already started. Gilberts’ appointment marks the foundation of Rothamsted Experimental Station (since January 2003 known as Rothamsted Research, and subsequently referred to as Rothamsted). The series of field experiments started between 1843 and 1856 are known as the ‘Classical’ Experiments and several of the original experiments continue today. The original purpose of the long-term experiments (LTEs), was to study the effects of fertilizer treatments on crop production. The experiments focussed on several crops, including winter wheat, spring barley, legumes, root crops and permanent grassland cut for hay. They are the longest running agronomic experiments in the world and are an invaluable and unique resource. The founding of the modern fertilizer industry was based on these original experiments^1^. Over time, they have been adapted with modifications in treatments, cultivars, and management to ensure they remain relevant to contemporary agriculture.

**Figure 1:**
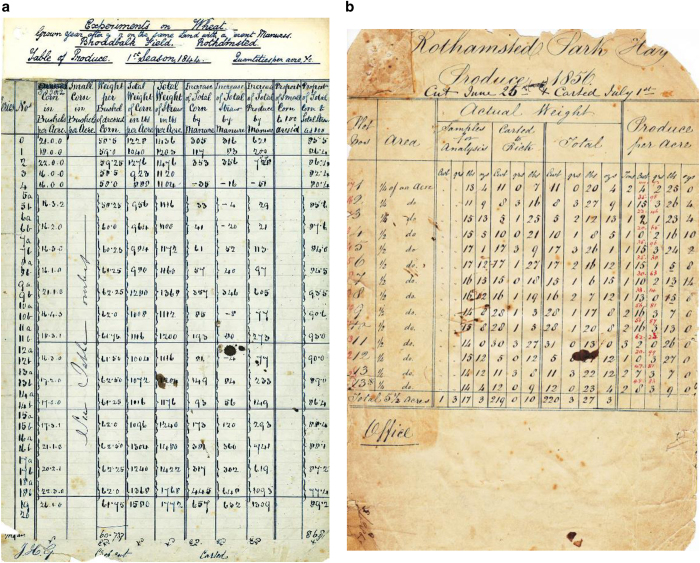
Historical record sheets. **a.** Broadbalk Yields 1^st^ Season 1844; **b**. Park Grass hay produce 1856 cut June 25^th^ carted July 1^st^.

Lawes and Gilbert had the forethought to archive samples of soil, grain, straw, vegetation and fertilizer, relating to their field experiments, together with data for crop and soil analyses and yields. They had realised that new analytical techniques were being developed and that, by retaining the samples, further opportunities for understanding might arise, for example the availability of nutrients in soils: “*But if our knowledge of the chemistry of soils should progress as rapidly as it has during the last twenty years, the analysis of a soil will ere long become much more significant than it is at present*”^2^. These physical samples, now comprising over 300 000 items, are kept in the Rothamsted Sample Archive (RSA; established 1843). They have been used by scientists from Rothamsted and across the world for retrospective analyses that would not have been possible when the samples were first taken^3,4^. Meticulous records were also kept of data relating to the LTEs, not only of crop yields, crop samples and soil properties, but also of other aspects such as herbage species composition, arable weed surveys and crop disease scores. In addition, qualitative agronomic information, such as sowing and harvest dates, amounts and dates of applications of fertilizer and manure were maintained. Originally, this information was kept in paper records, which are now relatively fragile and not readily accessible (Fig. 1). Since 2000 most data are in electronic format.

Increasing amounts of this extensive suite of data from Rothamsted’s long-term agricultural field experiments are now stored in the electronic Rothamsted Archive (e-RA), an online managed database for permanent and secure storage of data^5–7^ and accessed via the e-RA website www.era.rothamsted.ac.uk. Data from many of the handwritten paper records have been put into the e-RA database, and completing the collection is an important ongoing process. These LTE data, and those from the meteorological stations at Rothamsted, are part of the Long-term Experiments National Capability (LTE-NCG, 2012–2017 and now 2017–2022), funded by the UK Biotechnology and Biological Sciences Research Council (BBSRC). The LTE-NCG also comprises the RSA and many of the Rothamsted LTEs themselves, as well as the Rothamsted UK Environmental Change Network (ECN) sites at Harpenden, Hertfordshire and North Wyke, Devon (www.ecn.ac.uk). This paper describes the development, content, application and uses of e-RA demonstrating how sharing these data enables knowledge enrichment, not just at Rothamsted but also for the wider scientific community.

## Results

### Numbers of users and types of requests

**Figure 2:**
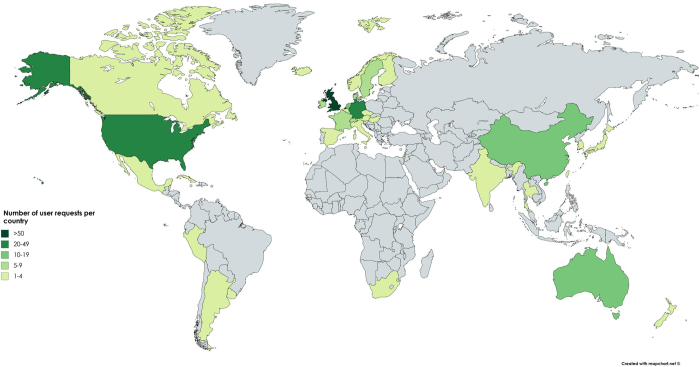
World distribution of requests for e-RA data (01/01/2010– 31/08/2017) where 289 of 571 UK users are external to Rothamsted Research.

**Figure 3:**
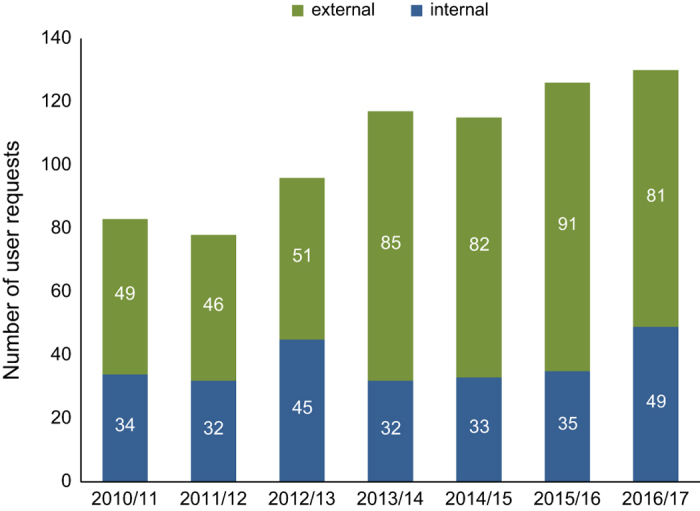
Numbers of e-RA user requests per academic year (1/09/2010-31/08/2017, internal Rothamsted staff (blue) and external (green) (totals: 2010/11–83, 2011/12-78, 2012/13-96, 2013/14-117, 2014/15-115, 2015/16-126, 2016/17-130).

Since the public launch in May 2013, e-RA has become an increasingly well-used and internationally recognised data resource for sustainable agriculture researchers, as illustrated by the global distribution of e-RA data users (Fig. 2). There is a wide range of research purposes given when data are requested (Table 1), with the top three categories of requests being crop and soil modelling, agronomy and agroecology. To date, almost 800 user requests for e-RA data have been received since monitoring of usage began in 2010. There has been a steady increase in the number of requests received annually (Fig. 3) and the increasing number of external users suggests that awareness of the potential value of the resource is growing. Most users learn of e-RA via a personal contact which means that users themselves find it useful and valuable and recommend it, or via web searches^8^.

**Figure 4:**
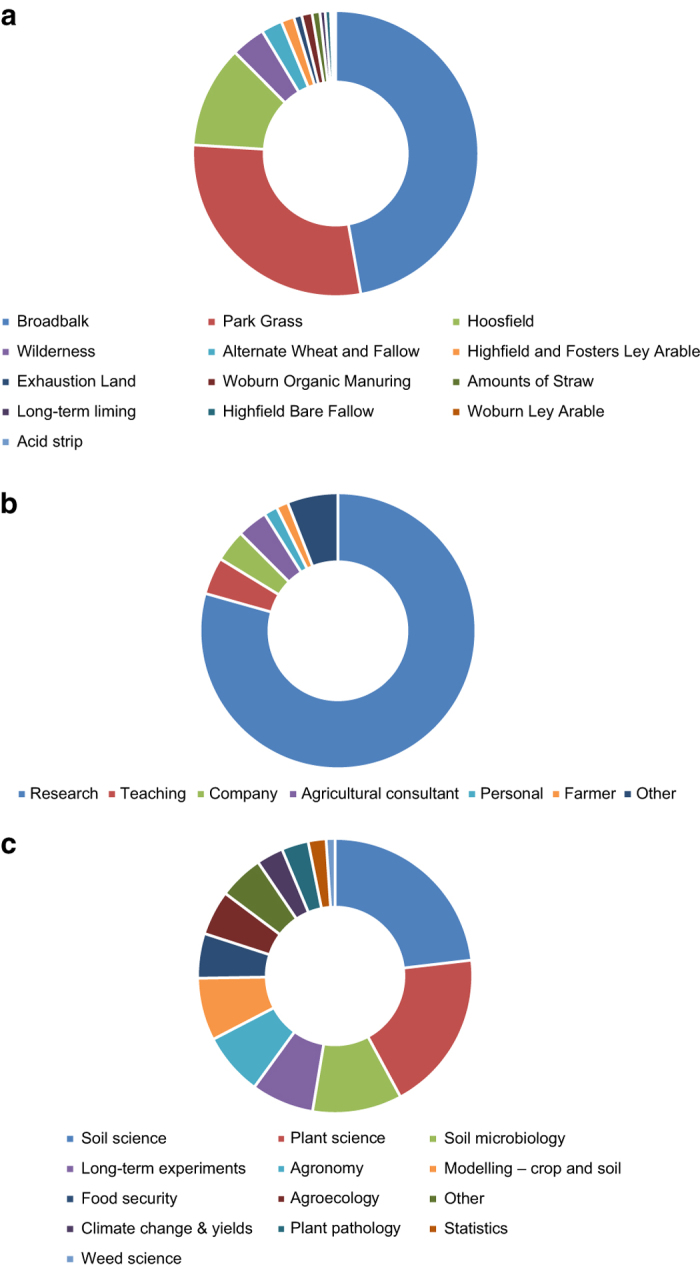
Requests for e-RA data and publications arising from the long-term experiments. **a.** Requests for e-RA data 01/01/2012-31/12/2016 *From a total of 535 (LTE 288 & Met 257) (External 356 & internal 179) -* which LTE; and **b.** Requests for e-RA data 01/01/2012-31/12/2016 *From a total of 535 (LTE 288 & Met 257) (External 356 & internal 179) -* which use; 4**c.** Publications linked to the Rothamsted LTEs (01/01/2012-31/12/2016); distribution per discipline.

Of the requests since the public launch in 2013, approximately half have been for data from the LTEs and half for meteorological data. Of the LTE data, most requests relate to the Broadbalk, Park Grass and Hoosfield experiments (Fig. 4a). The LTE variables most commonly requested are yields, soil properties (such as soil type and organic matter content) and experiment details/history. Of the meteorological data, most requests are for rainfall, air temperature and sunshine hours (Supplementary Figure 1). Most e-RA requests are from the research community in both universities and research institutes. Other users include university lecturers for teaching, conservation groups, agricultural consultants and companies (Fig. 4b). Approximately 80% of all users are from UK, 10% from Europe and 10% from outside Europe; a total of 30 different countries have requested data, 15 European and 15 from outside Europe; outside the UK, the USA has the greatest numbers of user requests, followed by Germany and the Netherlands (Supplementary Figure 2).

### Publications relating to the Long-Term Experiments

The scientific impact of the LTEs is demonstrated in part by the record of publications from research that uses the Rothamsted LTE and meteorological data, some of which is obtained through e-RA (not all research activities resulting in these publications will have used e-RA data, some use new data generated from fresh samples taken from the experiments themselves or from dried material taken from the RSA). Today there are an average of 18 publications per year (mean of last 10 years 2007–2016, range 5–27). Between 2007 and 2016 Broadbalk was cited in 81 papers, 55 cited Park Grass and 20 cited Hoosfield. In 2012–2016, most of the publications related to soil science, plant science, soil microbiology, crop & soil modelling as well as general papers on long-term experiments (Fig. 4c).

### Case studies using data from e-RA

The wide relevance of the Rothamsted LTEs to agriculture and environmental science research is illustrated by selected case studies below. These have been chosen to show the range and variation both in subject areas utilising the data, and in institutions using the data, nationally and internationally. These case studies have sometimes used e-RA data alone and sometimes in conjunction with samples from the sample archive or the current LTE experiments themselves.

#### Soil organic carbon (SOC) models

One of the most common requests for e-RA is for providing data to parameterize and/or validate computer models of crop production and changes in SOC in response to changes in management and climate. The different fertilizer treatments applied to the Rothamsted LTEs have resulted in a wide range of SOC contents and data from various LTEs have been used in developing and testing models of SOC dynamics, including the widely used Rothamsted Carbon Model, RothC^9–12^. Other SOC models have made use of the data in e-RA, including data from the Park Grass experiment and Geescroft Wilderness^13^, and the C-TOOL model was parameterized using data from Broadbalk and Hoosfield in addition to data from Danish and Swedish sites^14^.

#### Climate change and wheat yield predictions

Recent climate projections show that changes in temperature and precipitation may potentially affect UK crop yields. Broadbalk wheat yield data was used in calibration of the CERES-Wheat model and with regional model projections reported that increases in temperature generally lead to positive impacts on yield and a northward shift in cultivation^15^. The UK Climate Change Risk Assessment predicted 40–140% increase in wheat yield by 2050 in the UK based on a simple regression model linking yield changes with temperature increase^16^. Using Broadbalk data, it was demonstrated that this analysis had serious shortcomings and did not take account of key factors responsible for increased wheat yields over the preceding 50 years, and established that yield increases were due to high-yielding dwarf varieties, better pest and disease control and higher NPK fertilizer application^17^. Moreover, recent simulation of wheat yields by the multi-model ensemble of wheat models showed that global wheat production is estimated to fall by 6% for each ^o^C of temperature increase and become more variable over space and time^18^. The meteorological and crop yield data in e-RA are widely used as a resource for calibrating and testing crop models^19^.

#### Weed species dynamics

Various weed investigations have been conducted on the Broadbalk wheat experiment section 8 which has not received any herbicides during its 170-plus-year history. Annual non-destructive surveys have been done since 1991 using a standard protocol. Analysis of these species data (1991–2002) revealed long-term trends in weed frequencies and population differences between variously treated plots. Some species (e.g. common chickweed) significantly preferred increased amounts of nitrogen (N) fertilizer, whilst others were strongly disadvantaged (e.g., the legume, black medic and rare weeds such as corn buttercup and field horsetail); others showed little response to differing N rates (e.g., blackgrass and corn poppy)^20^. Changes in weed flora traits in response to increasing N inputs were examined and it was confirmed that assemblages dominated by rare/threatened species declined as fertility increased. The data were used to define a ‘rare weed traits syndrome’ of short stature, late flowering and large seed that has been selected against by the high nutrient inputs that characterise modern agriculture^21^. Another study demonstrated that certain pairs of arable weed species were adapted to similar fertilizer levels but diverged in their response to climate hence confirming the storage effect hypothesis and shedding light on how species with similar resource requirements co-exist at a temporal scale^22^. Finally, it is of note that the Broadbalk experiment is home to the last naturally occurring population of *Galium tricornutum* (corn cleavers), an extremely rare plant nationally.

#### Nutritional quality of grain

The Broadbalk experiment has enabled study of the effects of soil nutrient status and other factors on crop grain micronutrients. Selenium (Se) is a vital micronutrient, essential for human health and crops produced in many regions in the world are low in Se. The Se concentration of over 160 years of archived wheat grain samples from the Broadbalk experiment were analysed and it was shown that the Se concentration was influenced by sulphur (S) inputs from fertilizers and atmospheric deposition^23^. Furthermore, it was shown that not adding S fertiliser increases Se, and molybdenum (Mo) concentrations in wheat plant tissues, as a response to S deficiency^24^. Another study identified climate–soil interactions as main controlling factors of the Se concentrations in soil and that climate change is likely to increase Se deficiency in more than 60% of agricultural land world-wide^25^.

#### Grazing quality

Long-term soil and herbage samples from the Park Grass experiment were analysed for iodine (I) and Se retention in soil and subsequent uptake by herbage. The results from the variously treated plots were analysed to assess fertilizer, yield, soils chemistry and rainfall effects during 1876-2008. A growth-dilution effect for I and Se was suggested by the positive correlation between growing season, rainfall and herbage yield, and their concentrations were reduced if phosphate and sulphate fertilizers were applied. Results suggested that the iodine requirements of grazing animals are not likely to be met by herbage alone^26^.

#### Biodiversity of grassland species

Long-term records of species data from the Park Grass experiment presented a positive response of biodiversity to reducing N inputs from either atmospheric pollution or fertilizers. Diversity has increased over the past three decades on Park Grass plots that had received N fertiliser for over 130 years before it was withheld from 1989. Plant diversity on some plots is now at levels equivalent to that on plots which have never received N fertilizer. Diversity also increased over the same time-period more widely across the experiment as atmospheric N deposition decreased due to clean air policies. The authors acknowledge the global importance of the Park Grass Experiment because it acts as a ‘living barometer’ of the impact of environmental change on biological systems^27^.

#### Nutrient modelling

The Rothamsted Experiments offered an opportunity to test the N14CP model against long-term soil C, N and biomass time series, providing data on the responses of the plant-soil system to management and changes in atmospheric deposition, especially that of N. Selected unfertilised treatments from Park Grass and the Geescroft Wilderness experiments provided two contrasting land-use change tests of this model and showed that N deposition has a large effect on the latter whilst biomass removal has reduced the effect of N deposition on Park Grass^28^.

#### Genomics

The hypothesis that a key influence on plant biomass and species composition is the interaction between N and Phosphorus (P) availability and plant genome size (GS), was tested using data from the Park Grass experiment^29^. This study used data on species dry weight recorded over 10 years (1991–2000)^30^ and showed that the biomass-weighted mean GS of species growing on plots with the addition of both N and P fertilizer were significantly higher than that of plants growing on control plots and plots with neither N or P. The plants on these N+P plots are dominated by polyploids with large GS and a competitive plant strategy. If these findings are a general grassland phenomenon, then GS may be an important trait to consider in models predicting changes in plant community structure resulting from climate change or anthropogenic-induced perturbations to the environment, such as fertiliser run off.

#### Plant Physiology and climate change

Intrinsic water-use efficiency (W_i_) in grassland communities was demonstrated to consistently increase over a wide range of nutrient levels, soil pH and plant community compositions during the last century. The study used long-term yield data and species richness from e-RA and carried out C isotope analysis of archived samples from 16 contrasting fertilizer treatments, with and without lime. Meteorological data for the studies was also supplied by e-RA^31–33^.

#### Nitrate leaching

Nitrate leaching from Broadbalk (1990–1998) and its relation to weather and fertilizer treatment data, and interactions between them, was analysed^34^. The weather pattern was the dominant factor controlling N loss. Both the concentration of nitrate in the drainage waters and the amount of N leached increased with the amount of N applied, mostly because of long-term, differential increases in soil organic matter and mineralization. Losses measured 120 years ago, from identical treatments were 74% greater than current losses because of today's larger yields and more efficient varieties and management practices. Data from the Rothamsted meteorological station drain gauges and 1/1000^th^ acre rain gauge was used to determine long-term leaching losses of nitrate through bare, unmanured soil^35^.

#### Plant Pathology

Rothamsted meteorological data available in e-RA and archived samples of wheat grain and straw from Broadbalk enabled detection of changes over time in leaf blotch diseases of wheat to be investigated^36^. Long term variation in *Phaeosphaeria nodorum* and *Mycosphaerella graminicola* DNA over the period 1844–2003 was dominated by factors related to nationwide sulphur dioxide (SO_2_) emissions. Annual variability was dominated by weather factors over a period longer than the growing season. The results from this study confirmed the correlation of man-made SO_2_ emissions and meteorological factors with plant-pathogen interactions and emphasize the importance of long-term monitoring of air pollutants and climate change.

#### Insect pest forecasting

The Rothamsted Insect Survey (http://www.rothamsted.ac.uk/insect-survey/) has been operating traps for five decades and uses meteorological data from e-RA and others for the aphid forecasts published annually since the 1980s (Bell, J. Insect Survey, personal communication). This alert broadcasts news on the distribution and abundance of pest aphids at a regional scale that aids aphid control decisions. The data are used for fundamental studies on factors affecting the dynamics of aphid populations and they cite strong relationships between winter temperature and both the time that aphids are first found in traps, and their abundance^37^.

### Other uses of e-RA

These case studies highlight just some of the research that has used e-RA data but there are many more users requesting e-RA data including University teaching courses (e.g. University of East Anglia, Open University), statistics courses (University of Nottingham, Rothamsted Research), undergraduate projects (e.g. Bournemouth University, Plymouth University) and conservation groups (e.g. Herts and Middlesex Wildlife Trust). Furthermore, subsets of data are also available for schools’ use. More unusual requests have included historical agriculture e.g. spatial dynamical modelling to reconstruct and understand the development of the cultural landscape in the Dutch part of the Roman Limes river borders (VU University, Amsterdam http://limeslimits.wordpress.com/) and archaeobotany e.g. land-use strategies of prehistoric farmers in Germany and Switzerland (University of Basel, Switzerland). Thus, many of these uses of the LTEs were not foreseen at their initiation, and yet the original purpose, understanding the connection between crop production and soil fertility, is still relevant today.

## Discussion

Increasing value has been placed on long-term agricultural experiments as long-time series of data are essential to fully understand the effects of new crop management practices, industrial pollution and climate change on both the crop-soil system and the wider environment^3,38–40^. Interest in LTEs worldwide has increased exponentially and encouraged access to the data arising from them^41^. In addition, the significance of LTEs, such as those at Rothamsted, as a vital component of research to enable development of more sustainable agricultural systems to meet the challenge of increased food security has been highlighted^42,43^. In a recent example, options for increasing soil organic matter to help mitigate global warming have been investigated in work utilising soil carbon data from long-term experiments at Rothamsted to address the practicality of the ‘four parts per 1000’ climate change initiative^44^. As we reach 175 years since the inception of Rothamsted’s LTEs their value and relevance to agroecology, crop protection and sustainable agriculture endures.

Rothamsted Research’s long-standing commitment to making its LTE data available to the scientific community is shown by the investment that has been made to develop e-RA and provide a dedicated data management and curation team to support data storage and dissemination. Consequently, the Rothamsted LTEs have become an increasingly important resource and, facilitated by e-RA, they have advanced data sharing and knowledge generation. However, since the last major release of e-RA, data science and the technologies and standards for discovering, interpreting and linking data, particularly for automated machine discovery, have evolved. e-RA must now address this new landscape to ensure our data are readily findable, accessible, interoperable and re-usable. Therefore, the next phase in e-RAs development will include the adoption and implementation of FAIR data principles (see Future Developments).

Already there is a demonstrated demand for e-RA data. However, due to its long history, the LTE data has inherent complexities and researchers can require specialist support from the curators to understand and correctly use the data. To ensure sustainability it is essential we provide researchers with improved tools to independently find and select data. To do this we intend to provide better structured metadata, to employ improved visualisation tools to support researchers, and to improve semantic descriptors by using existing publicly available standards. We hope this next phase in e-RA’s development will enable more researchers and machines to discover our data and provide researchers with the means to better and more independently understand, identify and re-use appropriate datasets, ultimately enabling more scientists to benefit from this historical and unique data repository.

## Methods

### Development of e-RA

Plans for e-RA were laid out in 1990 and it was initiated in 1991 (ref. 45), funded by the Lawes Agricultural Trust (LAT) and the Leverhulme Trust. At this stage, it was based on an ORACLE database management (V5 then V7) system under the UNIX™ operating system and programs specific to e-RA were written to perform tasks of data entry, description and extraction. Perl scripts (Practical Extraction and Reporting Language https://www.perl.org/) were used to generate static content HTML pages^46^. A dedicated website for data extraction and background information was developed and this was one of the earliest data portal sites in the UK. The initial test version of the e-RA database Version 1 (V1) was available with a limited subset of data in 1993.

A major refactoring of the e-RA code-base began in 2005 and, following migration to Microsoft SQL Server, Version 2 (V2) of the e-RA database was released to Rothamsted users in February 2009. A new e-RA website www.era.rothamsted.ac.uk was released in March 2011, incorporating an extensive overhaul of the previous site with major additions to the content including comprehensive background information about the field experiments and meteorological data.

Refinements were made to the database to provide public access to an updated e-RA V2 and the launch of the e-RA database externally to the wider scientific community (May 2013). This is accessed using the e-RA data extraction tool (DET) via the e-RA website. It is accompanied by extensive supporting documentation and the e-RA curators provide tailored assistance and support in selecting and understanding data as suits users’ needs.

### Entering Data in to e-RA

#### Collation and curation

**Figure 5:**
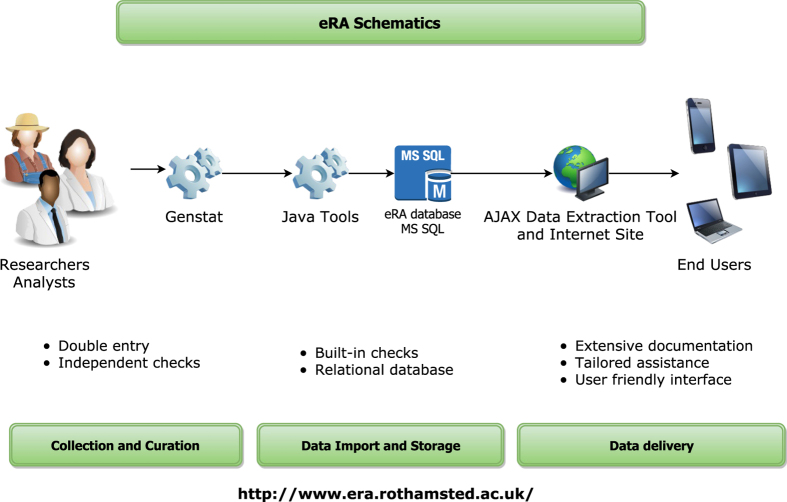
e-RA schematics illustrating the process for archiving the long-term data from source to database.

The process for capturing data in e-RA from the LTEs is multi-staged (Fig. 5). Data are provided by the Rothamsted farm staff and research scientists who have analysed the samples. The data is then checked and formatted by statisticians using data conditioning programs written in GenStat^47^. This checked data is then directed to the e-RA curators who perform independent quality checks. It is uploaded into e-RA and stored in a Microsoft SQL Server database. Since 2000, much of the data generated each year is in electronic form and the procedures for data entry and validation have changed. The electronic data is sent to a designated statistician for compilation prior to checking and formatting in GenStat. The GenStat outputs are further checked by researchers and the e-RA data curators. Any apparent anomalies in the data are investigated and resolved prior to archiving.

#### The database structure and e-RA programs

The data in e-RA are divided into DATASETS corresponding to sets of related observations, e.g. yields for the Broadbalk Wheat Experiment. The concept behind the approach used in e-RA anticipates there may be changes in the way data are collected over time and thus one or more subsets are needed, termed SHEETS to accommodate such historical changes. Each sheet may in turn be composed of GROUPS corresponding to a group of data in the original paper record. Each group is a collection of data fields, the table columns which hold the data. Different sheets represent different historical periods of the experimental record, whilst different groups within the same sheet refer to different sets of data for the same historical period. This is primarily useful for data entry. Sheets of data are presented to e-RA with a corresponding data definition contained in a Header File. The Header File contains technical metadata for the data file and includes layout and format of the data and variable name definitions. There may be many hundreds of sheets for one dataset. Different sheets allow for the variation in layout and format, which varies across experiments and with time as the experiments have evolved. Using Header Files enables the associated data files to be captured in a format which resembles the original sources and records as closely as possible while mapping to a common data representation in the database. Furthermore, e-RA Header Files provide a range of validation checks to be specified within a dataset, such as expected ranges for the variables and other specific methods to check accuracy and consistency. Once the sheets and their descriptions have been prepared, the curators use a suite of Java™ programs to upload them to the database. The programs update the metadata held in the e-RA database for the dataset and append the new sheet to the original dataset. A dataset in e-RA is represented in one single table. The e-RA custom code is not currently available.

### The process for Data Users

Data stored within e-RA are freely available to the international research community and other interested parties via the internet. Access to data is granted after registration which requires brief details of the data required, of how the prospective user became aware of e-RA (for monitoring and future publicity reasons) and the completion of a Data Access Policy (DAP) agreement. The DAP details the scientific, or otherwise, case for obtaining data, the requesting organisation, brief details of the research project for which the data will be used, and the specific details of the data requested, including which experiment(s), years and plots/treatments. The users are requested not to pass the data on to a third party and asked that they acknowledge Rothamsted Research as the source of the data in any publications. If the user’s research request is particularly complex or the datasets in e-RA are known to have difficult structural problems (e.g. changes in the experiment), the user will be given further support from one of the database curators or if appropriate be offered the opportunity to work with a Rothamsted scientist who might be a suitable collaborator. The user is then given a password-protected account to extract the data, using the data extraction tool (DET) accessed via the e-RA website.

All information collected about user requests and how the data were provided is maintained in a confidential Microsoft Access database. This utilisation data is used (anonymously) to provide monitoring data regarding e-RA usage to our funding agency. As outlined in their Data Sharing Policy^48^ the BBSRC requires grant holders to record where and how data have been shared. We collect this data to evidence the impact of the LTEs and justify continued National Capability funding.

When using the DET, the user selects the dataset and then specifies the precise data requirements by selecting and filtering by section, plot, year, or any other variable available for that dataset. The user can specify the sort order of the fields and then data are extracted and delivered in a separate window. From there, data can be copied and pasted into a spreadsheet for subsequent analysis or extracted as a CSV file. Online contextual help is provided in the DET. In the case of the meteorological data, additional calculated values (e.g. evapotranspiration rate) from the raw data are provided within the DET for convenience.

While some users will want to have a login access to e-RA and source their own data, most users who contact the e-RA team opt to have the relevant dataset appropriate for their research extracted on their behalf. This is especially appropriate if it is a very complex request. However, in either data retrieval scenarios, full support and advice is provided by the e-RA curators in terms of the most appropriate and relevant data to be extracted from the database for the user’s purposes and in its interpretation and understanding.

#### Previews of data

Data sub-sets are presented as previews for each of the complete datasets. This allows a user to examine the data prior to registering, enabling determination of whether the data is of interest and relevance. These previews are available as downloadable Excel files on the relevant web pages of each field experiment.

### Content of e-RA database

#### Long-term Experiments (LTEs) data

The e-RA database currently has over 30 datasets (Table 2) containing records of yields and other data, such as botanical composition, relating to five of the original Classical Experiments: Broadbalk Winter Wheat, Park Grass Continuous Hay, Hoosfield Spring Barley, the Alternate Wheat and Fallow and the Broadbalk and Geescroft Wilderness sites, as well as the Woburn Ley-arable experiment (started 1938). The Broadbalk datasets also include yields from the other rotational crops introduced since 1968 (potatoes, beans, maize and oats). There have been several changes in experiment management over time, including plot divisions, the introduction of new cultivars, fertilizers and treatments. These modifications have taken place to ensure that the LTEs remain relevant to current agricultural practice and/or environmental issues, whilst maintaining the long-term integrity of the experiment, and thus datasets are often broken up into subsets relating to significant management changes. For example, Broadbalk yields are presented in four datasets relating to changes in the experimental layout and treatments which have occurred over time (Supplementary Figure 3). The changes in the treatments, layout and plot nomenclature of just one plot (Plot 2) on the Park Grass Experiment in response to increasing soil acidity is illustrated and documents the increasing complexity of the experiment over time (Supplementary Figure 4 and Fig. 1^49^).

There have also been changes in how the LTEs were managed. For example, a change to the harvest method on Park Grass in the 1960s (ref. 26) and changes to soil and crop analysis techniques^50^. Some variables/datasets are available for the duration of the whole experiments, e.g. yields, and some are for shorter periods e.g. Broadbalk grain quality from 1974 to current, and other datasets for a discrete time-period, e.g. the Park Grass botanical survey from 1991–2000 or for a part of the experiment, e.g. the 1991–2014 weed surveys relate to Broadbalk Section 8, the no-herbicide section. These historical changes to the layout, treatments and management of the LTEs require a thorough understanding of each experiment before results can be rigorously analysed and interpreted correctly. The e-RA curators together with the LTE researchers provide this understanding and expertise enabling users both to access the best datasets and to understand these complex experiments and their data.

#### The meteorological data

Meteorological data from Rothamsted are also held in the e-RA database (Table 3). The earliest meteorological variables in e-RA are rainfall and wind direction at Rothamsted, Harpenden, measured from 1853. Meteorological data are also available from two other Rothamsted Research sites, Woburn (Bedfordshire), since 1928 and Broom’s Barn (Suffolk) since 1982, though fewer variables are measured than at Rothamsted, Harpenden. Variables were measured manually each day at 9 am, until 2004 (1999 for Woburn) when the systems were automated. Some manual observations continued to be recorded at Rothamsted (e.g. cloud cover) until 2007. Some variables are continuous e.g. air temperature to the present day and others are for discrete periods of time e.g. depth of fresh snow 1960–78. Also available are two simplified Rothamsted meteorological datasets specifically designed for schools.

All the standard meteorological measurements are made at Rothamsted, plus some extra measurements unique to the site. These include drainage from three ‘drain gauges’ constructed at Rothamsted in 1870. These consist of undisturbed blocks of soil 20, 40 and 60 inches deep (51, 102 and 152 cm, respectively), which are never cropped and are kept free of weeds, originally by hand weeding and subsequently using herbicides^35^. Also, unique to Rothamsted is rainfall measured from the 1/1000^th^ acre rain gauge (4.047 square meters), constructed by Lawes in 1852/3. This is used in conjunction with the three drain gauges, which have the same surface area. Derived meteorological variables are available including the Potential Soil Moisture Deficit (PSMD, cumulative value) and day degrees above (DDA,) or below (DDB,) a base temperature.

Meteorological measurements and observations from the Rothamsted, Woburn and Brooms Barn weather stations have been used by the Meteorological Office (MO) since 1878, 1959 and 1964, respectively. In May 2017, Rothamsted was recognised by the World Meteorological Organisation, nominated by the UK Meteorological Office, as a Global Long-term Observing Station that has been providing observations for over 100 years. Rothamsted air temperature data from 1878 onwards is also an important part of the long running Central England Temperature (CET) series, a series of homogenized daily values representative of Central England^51,52^.

For a complete description of the field experiments and weather station history see the Rothamsted Guide to the Long-term Experiments^53^ and the e-RA website www.era.rothamsted.ac.uk.

#### Future content

The e-RA database is an evolving resource, growing with every year of operation and responding to the needs of scientists at Rothamsted and elsewhere. Annually collected data (yields, weed surveys, etc.) are added each year following QA and statistical analysis and meteorological data are added daily. Additional historical data will be added to the datasets for the existing LTEs, including more soils and crop nutrient data. Data from other LTEs including the Exhaustion Land experiment and the Woburn Organic Manuring experiment will also be added.

### Open Access Data

**Figure 6:**
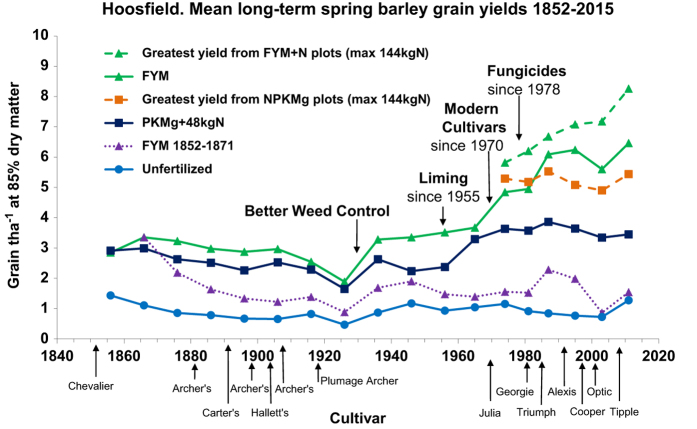
Hoosfield Open Access chart. Mean long-term spring barley yields, 1852–2015 https://doi.org/10.23637/KeyRefOAHByields.

Selected datasets from the long-term experiments are available as open access data with no requirement for user registration. These have been developed from commonly requested subsets or summaries of data (Table 4) and are available for direct download from the e-RA website in the form of charts and excel data sheets. Generally, these datasets comprise data already published, for instance the Broadbalk yields Open Access dataset^54,55^ whereas others are previously unpublished data (e.g. Fig. 6). Simplified subsets of Rothamsted meteorological data for use in schools (Table 5) are also available to directly download as excel files. The e-RA curators are progressively adding to these Open Access datasets.

### Background and supporting information

Alongside the curated datasets in e-RA there is an extensive collection of qualitative and descriptive information relating to the experiments, both historical and current. This is vital for interpretation of the data, and is made available in the e-RA website. These include current and historical treatment and management practices, field plans, farm maps, plot sizes, soil maps, soil descriptions, cultivars, sowing dates, harvest dates, methodology, cropping rotations and aerial photographs of field experiments together with links to key references as well as photographs of the farm, the experiments and the equipment.

### Bibliography

The e-RA bibliography (http://www.era.rothamsted.ac.uk/papers) is a comprehensive database of research publications related to the Rothamsted LTEs. It currently contains over 1,600 references, searchable by author, title, experiment and year, and includes abstracts. Where available, links are provided to the original papers (for example, via DOI or through our internal electronic publication repository eRAdoc, see below). These are regularly updated with information sourced from Web of Science alerts or when data users inform the e-RA curators that LTE data has been used in a publication, one of the conditions specified in the Data Access Policy. Being able to cross-reference publications with the source datasets in this way is an important source of information both for other researchers and for the e-RA curation team to monitor use and application of the LTEs.

The bibliography includes over 500 references to publications by Lawes and Gilbert between 1842 (ref. 56) and 1900 (ref. 57). These encompass a wide range of topics in addition to the Rothamsted LTEs, including the disposal of sewage and the nutrition of farm animals. Not included are references to the many letters, comments or reports of meetings where Lawes or Gilbert are only mentioned or quoted. For the complete list of Lawes and Gilbert publications, see Dyke^58^.

### Rothamsted’s historical document repository - eRAdoc

eRAdoc (http://www.era.rothamsted.ac.uk/eradoc) is an online repository for Rothamsted’s historical documents that relate to the LTEs. It comprises Rothamsted Annual Reports, guides, yield books, maps and plans, and other documents relating to the LTEs printed at Rothamsted since the 19th century. These documents were previously only available as paper copy. The Rothamsted Annual Reports, published since 1909, are a vital source of information about the LTEs^59–61^. Until 1988 they included data and important original articles about the LTEs, which were not published elsewhere. eRADoc delivers searchable PDF versions of the books with the added value of comprehensive tables of contents and the facility to download or link to specific articles or portions of books. It is the most recent resource within e-RA and relevant historical documents are still being added.

### Open Access Policy and Future developments

The purpose of e-RA is to store, communicate and disseminate LTE data for re-use by the wider scientific community. Since the last major revision of the e-RA website in 2013, open access publication of scientific data has come to be expected by funders, publishers and the wider community^48^. In parallel, new standards and technologies for supporting dataset discovery and linkage have been developed. The next phase for e-RA will look to adopt FAIR data principles^62^ and apply semantic and linked data technologies.

A first step has been to update Open Access summary datasets, already published on e-RA, (Table 4) with Creative Commons Attribution 4.0 International Public Licences, assign DataCite (https://www.datacite.org/) DOIs to them and provide structured metadata descriptions. The data are currently available as PDF images and annotated Excel spreadsheets, a next step will be to provide a JSON format which can be visualised as a dynamic chart using javascript or converted to Excel or CSV formats. Similar improvements will be made to the datasets available via the DET, however, to monitor dataset usage for reporting to funders, these will remain behind the e-RA registration wall. We will review the registration process, alongside reported DOI citation metrics, to ensure researcher access to data is as seamless as possible. We anticipate tracking and reporting DOIs using Altmetrics (https://www.altmetric.com) which generate alternative measures of scientific impact, and will support efforts to monitor usage of the experiments and datasets.

As part of our plan to apply FAIR data principles to our data, we are in the process of upgrading the discovery metadata descriptions for the rich catalogue of supporting material needed to interpret the data, including the historical plot maps and treatment, cultivar and rotation schedules described above. Our objective is to help researchers identify which of these resources are relevant to the datasets they are interested in. To address this, we will use DOIs for supporting material in the DataCite metadata schemas to map datasets to the resources necessary for their interpretation. Assigning DOIs to LTE datasets and supporting materials will enable researchers to formally cite their use in publications and provide a mechanism for acknowledging the contributions made by individual staff such as data curators and data managers.

To support interoperability with other agricultural datasets we are seeking to add semantic annotations to our existing datasets and express metadata for experiment features, such as site, environmental conditions, plot descriptions and experiment design using, and where appropriate, adding to existing ontologies, such as AGROVOC (http://aims.fao.org/standards/agrovoc/linked-data). From an initial review of data standards (ICASA Version 2.0 (ref. 63); https://data.lter-europe.net/deims/), checklists (MIAPPE^64^) and ontologies (Crop Research Ontology http://www.cropontology.org; Plant Experimental Conditions Ontology http://purl.bioontology.org/ontology/PECO) we have a strong body of existing resources for mapping our requirements for characterising LTE features and representing the LTE data. We are keen to work with the wider global network of long-term agriculture experiments to develop a recommended minimum checklist for LTEs and LTE data based on these resources. Furthermore, we hope by doing this to include LTE data as part of the increasing number of semantically linked datasets and resources such as the Springer Nature data portal, Scigraph (https://scigraph.springernature.com/explorer) and contribute to international efforts such as GODAN (Global Open Data for Agriculture and Nutrition http://www.godan.info/) promoting open agriculture data to address the global challenges facing food security.

## Additional information

**How to cite this article:** Perryman, S. A. M. *et al.* The electronic Rothamsted Archive (e-RA), an online resource for data from Rothamsted's long-term experiments. *Sci Data* 5:180072 doi: 10.1038/sdata.2018.72 (2018).

**Publisher’s note:** Springer Nature remains neutral with regard to jurisdictional claims in published maps and institutional affiliations.

## Supplementary Material

Supplementary Information

## Figures and Tables

**Table 1:** Disciplines for which the data available in e-RA are being used (01/01/2010-31/12/16).

**Project categories**	**Numbers of user requests**
Crop & soil modelling	108
Agronomy	70
Agroecology^a^	70
Soil Science	39
Crop Science	29
Other	25
Water, rivers, soil water	23
Education – schools and universities	16
Climate Change	14
Meteorology	14
Historical	13
Plant Pathology	11
Economics	5
Statistics	2

^a^including biodiversity, butterfly conservation, plant & insect ecology.

**Table 2:** Summary of Long-term Field Experiments datasets available in e-RA (September 2017)

**Data available**	**Dates available**	**Number of values ‘plots’ = plot x year**
**Field experiment name; crop grown and year established**		
**Broadbalk: winter wheat since autumn 1843; wheat in rotation with other crops since 1968**		
Wheat yields (grain & straw)	1844-present	>15,500 grain yields
Field bean yields (grain & straw)	1968-1978	209 grain yields
Potato yields (tubers)	1968-1996	551 tuber yields
Oat yields (grain & straw)	1996-present	342 grain yields
Maize yields (forage)	1997-2017	323 forage yields
Weed surveys:		
Whole experiment	1933-1979	>5,500 plots
Section 8 (no herbicides)	1991-2014	360 plots
Wheat root and stem diseases(take-all, brown foot rot, eyespot & sharp eyespot)	1968-2009	>900 plots
Crop nutrient content (% N, P, K, Ca, Mg, Na, S):		
Wheat grain & straw	1968-2013	>2,500 plots
Bean, potato, oat & maize	1968-2013	>1,200 plots
Grain quality (TGW, hectolitre weight, Hagberg falling number, grain size classes):		
Beans	1974-1978;	95 values
Oats	1996-present	>300 values
Wheat	1974-present	>8,000 values
Soil properties (%N, %SOC, pH, Olsen P, exchangeable cations, soil weight, CaCO_3_)	1865-2012 (13 sample years; not from all plots every year)	
**Hoosfield: spring barley since 1852**		
Barley yields (grain and straw)	1852-present	>10,000 grain yields
Grain quality:		
TGW and hectolitre weight	1974-present	>3500 values
Crop nutrient content (% N, P, K, Ca, Mg, Na):		
Barley grain and straw	1964-2010	>16,000 values
**Park Grass: continuous grassland since 1856, usually cut twice a year**		
Herbage yields (2 cuts)	1856-present	>16,000 yields
Botanical composition surveys (visual surveys & by weight)	1862-19761991-2000	>1,000 plots>1,100 plots
Insect surveys (Leafhoppers)	1977-1978(5 samples per year)	52 plots
**Alternate Wheat and Fallow: 1856-2015**		
Wheat yields (grain & straw)	1856-2015	311 grain yields
**Woburn Ley-arable experiment: Arable rotations with and without leys since 1938**		
Crop yield	1938-2007	280 values (Block III only)
Soil % organic Carbon and Nitrogen	1938-2009	192 values(Blocks I – V)

**Table 3:** Long-term daily (recorded at 9am GMT) meteorological data available in e-RA recorded at: Rothamsted from 1853; Woburn from 1928; Broom’s Barn from 1982.

Variable		**Rothamsted, Harpenden, Hertfordshire 51.82N 0.37 W 128 m asl**		**Woburn, Husband Crawley, Bedfordshire 52.02 N 0.62W 89 m asl**		**Brooms Barn, Higham, Bury St Edmunds, Suffolk 52.27 N 0.57E 70 m asl**	
	**Unit**	**Start**	**End date**	**Start**	**End date**	**Start**	**End date**
Rainfall	mm	1853	current	1928	current	1982	current
Rainfall 1/1000^th^ acre gauge	mm	1853	current				
Wind direction	0-360^o^	1853	current	1928	current	2012	current
Drainage from 20, 40 and 60-inch rain gauges	Inches	1870	current				
Maximum temperature	^o^C	1878	current	1928	current	1982	current
Minimum temperature	^o^C	1878	current	1928	current	1982	current
Sunshine	hours	1890	current	1928	current	1982	current
Grass minimum temperature	^o^C	1909	current	1929	current	1982	current
Dew point (derived)	^o^C	1915	current	1968	current	1982	current
Cloud cover	Oktas	1915	2007	1928	1999		
Barometric pressure	mb	1915	2003^a^	1928	1999^a^		
Wet bulb temperature	^o^C	1915	2014	1928	2009	1982	2009
Dry bulb temperature	^o^C	1915	current	1928	current	1982	current
Wind force/speed	msec^−1^	1915/1960	current	1928/1968	current	2012	current
Visibility	code	1923	2007	1928	1999		
Relative humidity (derived)	%	1925	2014	1928	2009	1982	2009
Relative humidity (recorded)	%	2014	current	2009	current	2009	current
Soil temperature under grass, various depths	^o^C	1931	current^a^	1928	current^a^	2012	current
Soil temperature under bare soil, various depths	^o^C	1931	current	1968	current	1982	current
Rainfall duration	hours	1931	current	1988	1999		
Total solar radiation^b^	MJ m^−2^	1931	current	1981	current	1982	current
Wind run	km	1946	current	1968	current	1982	current
Vapour pressure (derived)	mb	1946	current	1928	current^a^	1982	2009
Days with hail occurring	code	1960	2007^a^	1968	1999^a^		
Days with thunder occurring	code	1960	2007^a^	1968	1999^a^		
Days with snow occurring	code	1960	2007^a^	1968	1999^a^		
Depth of fresh snow	mm	1960	1978	1968	1978		
Total depth of snow	mm	1960	2007	1968	1999		
Days with fog occurring	code	1960	1978	1968	1978		
Snow lying	code	1960	1978	1968	1978		
State of ground	code					1982	1996
Net solar radiation	MJ m^−2^					1997	current
Daily met data for schools	6 variables	1990	current				
Monthly met data for schools	4 variables	1878	2013				

^a^Data not measured for all years

^b^J cm^−2^ at Rothamsted.

**Table 4:** Data available* as Open Access Datasets in e-RA, September 2017 http://www.era.rothamsted.ac.uk/open_access

**Dataset**	**Description**	**Dates**	**Comments**
**Broadbalk: winter wheat since autumn 1843: wheat in rotation with other crops since 1968**			
Broadbalk Yields^(Data Citation 1)^	Mean long-term wheat yields from selected treatments	Continuous wheat: 1852–2016 Wheat in rotation: 1968–2016	Widely used to demonstrate effects of FYM and fertilizers, improved cultivars and pest control on yield
Broadbalk soil organic carbon (SOC)^(Data Citation 2)^	Long-term changes in SOC in topsoil (0-23 cm) from selected treatments	Continuous wheat sections only: 1843–2010	Includes % SOC, measured soil weights and calculated SOC, tha^−1^
Broadbalk soil Olsen P concentration^(Data Citation 3)^	Long-term changes in Olsen P in topsoil (0–23 cm)	1843–2010, selected treatments	A measure of plant-available P
**Hoosfield: spring barley since 1852**			
Hoosfield Yields^(Data Citation 4)^	Mean long-term barley yields from selected treatments	1852–2015	Shows long-term changes in yield due to improved cultivars, pest control, FYM and fertilizers.
Hoosfield SOC^(Data Citation 5)^	Long-term changes in SOC in topsoil (0-23cm) from selected treatments	1852–1998	Includes % SOC, measured soil weights and calculated SOC, tha^−1^
**Park Grass: permanent grassland since 1856, usually cut twice a year**			
Park Grass species diversity^(Data Citation 6)^	Effect of selected fertilizer treatments, with and without chalk, on number of species	1864–2011	Number of species comprising 1% or more of the above-ground biomass.
Park Grass soil pH^(Data Citation 7)^	Long-term changes in soil pH (in water) from selected treatments	1856–2011	Shows acidification of soil due to fertilizer use and atmospheric deposition, and effect of liming
**Wilderness sites: two contrasting former arable sites which reverted to woodland in the 1880s**			
Broadbalk Wilderness accumulation of organic carbon in soil and trees^(Data Citation 8)^	Accumulation of biomass on a calcareous soil left to revert to woodland since 1882	Broadbalk Wilderness: 1881–1999.	Data on t C ha^−1^ accumulated in soil, litter, root and above-ground biomass (trees)
Geescroft Wilderness accumulation of organic carbon in soil and trees^(Data Citation 9)^	Accumulation of biomass on an acidic soil left to revert to woodland since 1883	Geescroft Wilderness: 1883–1999.	Data on t C ha^−1^ accumulated in soil, litter, root and above-ground biomass (trees)
**Rothamsted meteorological data**			
Mean annual temperature at Rothamsted^(Data Citation 10)^	Mean annual air temperature and five-year mean.	1878–2013	The mean annual temperature is now 1 °C higher than the 1878–1987 average.

^*^as Charts & Excel files

**Table 5:** Subsets of Open Access e-RA data available$ for schools www.era.rothamsted.ac.uk/Met/schools

**Dataset**	**Description**
**One month of daily weather**	Rothamsted July 2012
**130 year mean temperature**	5 and 10 year mean long-term temperature for Rothamsted
**Mean monthly weather January-December**	30 year means for three sites
**One year of monthly means**	2012 for two sites
**Daily rainfall for one month**	July 2015 for three sites

^$^as Excel files
