# Supplementary material for: The electronic Rothamsted Archive (e-RA), an online resource for data from the Rothamsted long-term experiments
Source: Sci Data. 2018 May 15;5:180072. doi: 10.1038/sdata.2018.72 (PMC5952867; doi:10.1038/sdata.2018.72)
Supplement: Supplementary Information [file sdata201872-s1.pdf]

Supplementary Information

Table of contents:

Supplementary Figure 1. Distribution of requests for e-RA data (1/1/2012-31/12/2016) – type of data;

Reference page 3.

Supplementary Figure 2. Distribution of requests for e-RA data (1/1/2012-31/12/2016) – source of request; Reference page 3.

Supplementary Figure 3. Broadbalk layout changes over time (schematic diagram not to scale); Reference page 10.

Supplementary Figure 4. Park Grass plot 2 changes over time (schematic diagram not to scale); Reference page 10.

a.

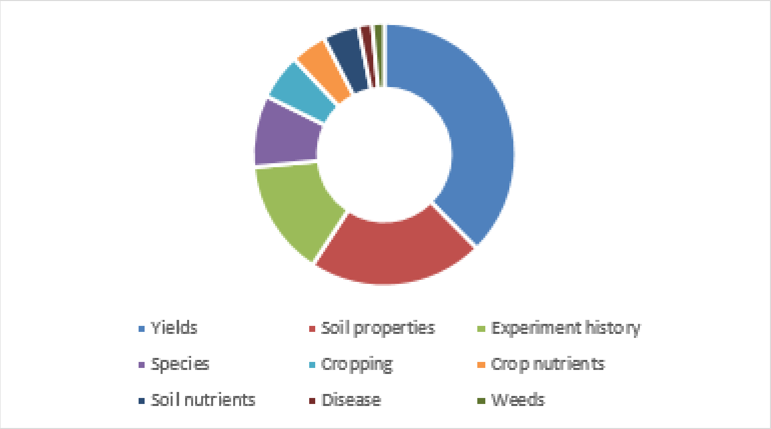

b.

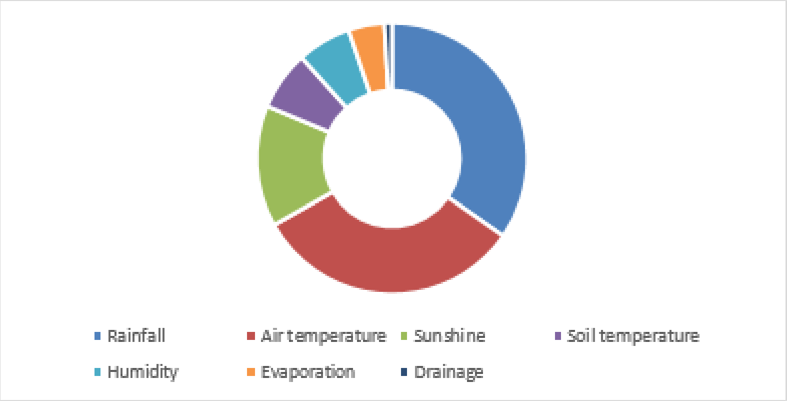

Supplementary Figure 1. Distribution of requests (internal and external) for e-RA data (01/01/2012-31/12/2016) – type of data: **a.** Variables from LTEs; **b.** Weather variables.

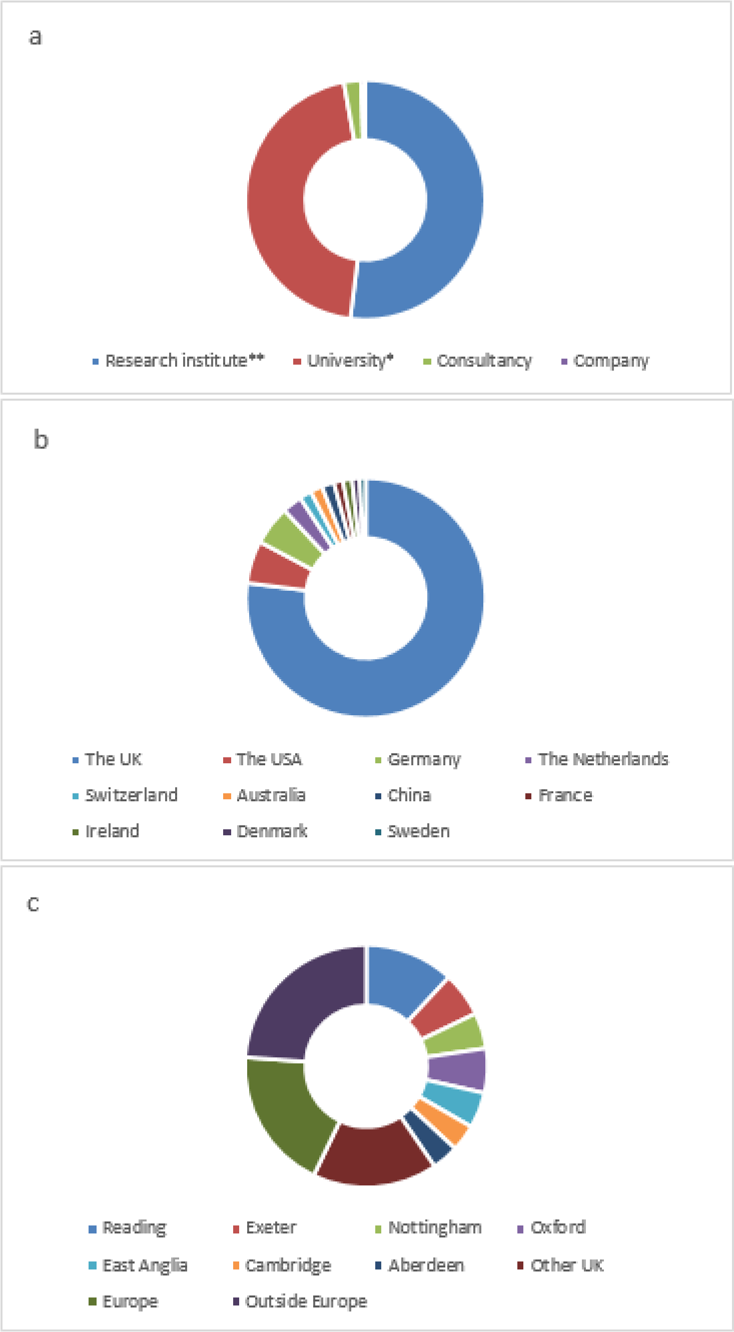

Supplementary Figure 2. Distribution of requests (internal and external) for e-RA data (01/01/2012-31/12/2016) – source of data request: **a.** Institute type. **b.** Country location of user; **c.** Universities requesting data.

a 1852-1925
BKYIELD 1844-1925

b 1926-1967
BKYIELD_F 1926-1953
BKYIELD_F85 1954-1967

c 1968-present
BKYIELD_R85 1968-2016>

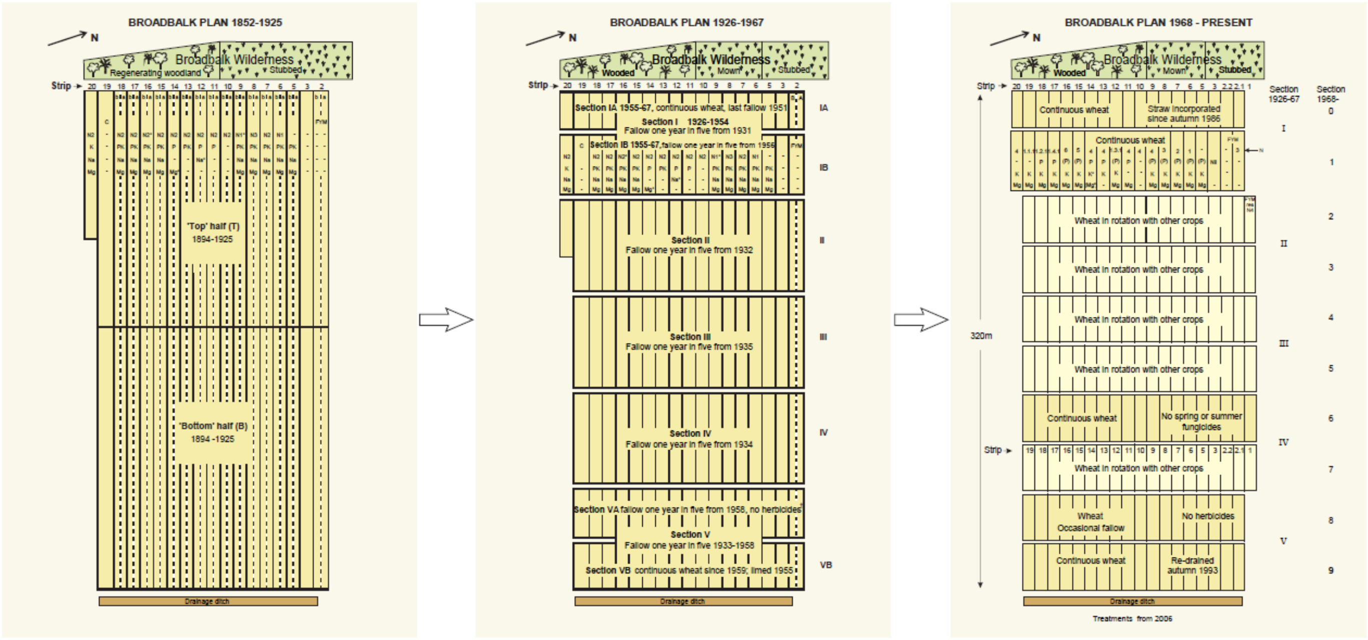

Supplementary Figure 3. Broadbalk layout changes over time (schematic diagram not to scale) **a**. Most strips divided into two halves lengthways (a and b) 1847-1893; then a and b halves combined to make one strip in 1894. Strips divided into top (T) and bottom (B) halves most years 1894-1925. Wilderness area had last wheat sown in autumn 1881; divided into two halves in 1900 and one remained as regenerating woodland, in the other half all woody species were removed ('stubbed') each year, to allow open ground vegetation to develop. **b.** The strips were sub-divided into five sections(I-V) in 1926. These were bare-fallowed sequentially to control weeds. In 1955 Section I was divided into two; IA received herbicides as required and was no longer fallowed, IB continued in the five-year cycle. In 1955 Section V was divided into two; VB received a single application of lime, VA did not. In 1963 Section VB was no longer fallowed and received herbicides as required, while VA continued in the five-year fallowing cycle. In the Wilderness area in 1957, the stubbed section was divided into two, one half remains 'stubbed', the other half was mown for three years, grazed by sheep each year from 1960-2000, and has been mown each year from 2001 (herbage not removed). **c.** Sections I-V were divided to create ten new sections (0-9) to allow the yield of wheat grown continuously to be compared with that of wheat grown in rotation after a two-year break.

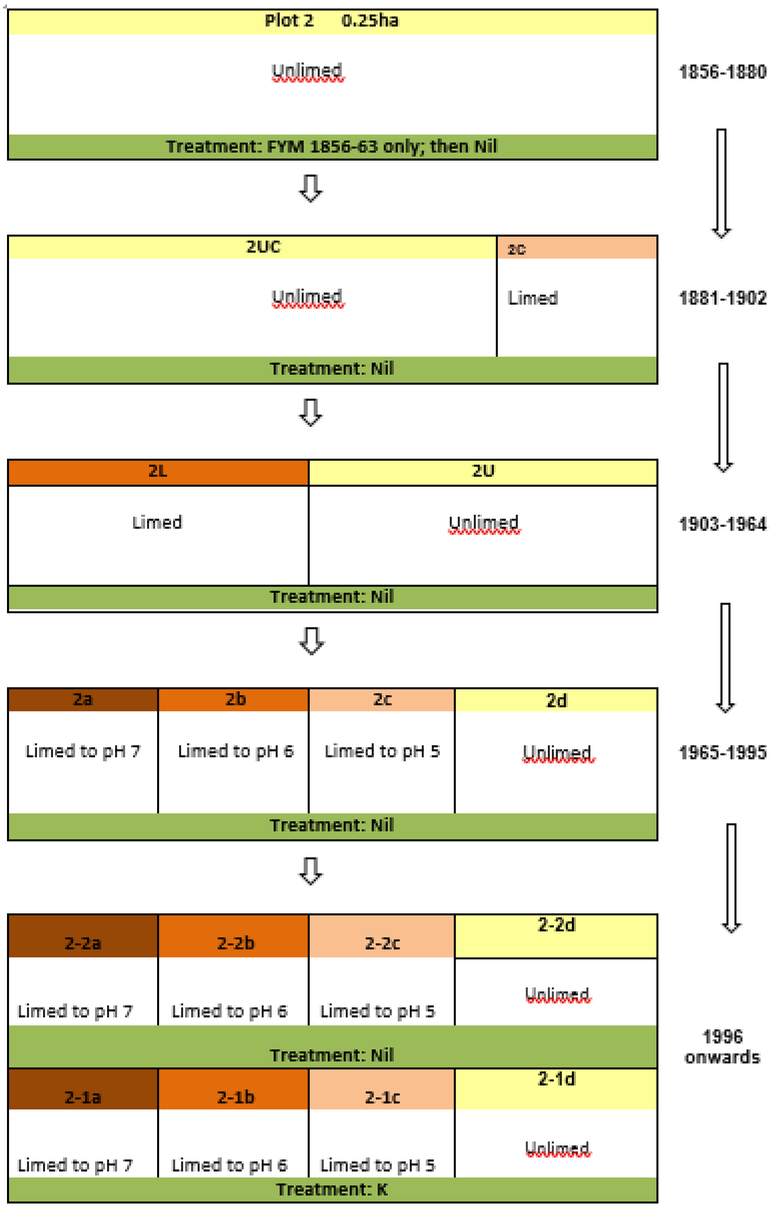

Supplementary Figure 4. Park Grass plot 2 changes over time (schematic diagram not to scale) (ref. p13). The experiment was established in 1856 on land that had been permanent pasture for at least 100 years; in 1903 plots were halved to test the effects of regular applications of lime; in 1965, plots were sub-divided into four sub-plots to apply chalk to maintain certain pH levels.
